# Response of Maize Seedlings to Silicon Dioxide Nanoparticles (SiO_2_NPs) under Drought Stress

**DOI:** 10.3390/plants12142592

**Published:** 2023-07-08

**Authors:** Asmaa A. Sharf-Eldin, Khairiah Mubarak Alwutayd, Ahmed Abou El-Yazied, Hossam S. El-Beltagi, Basmah M. Alharbi, Mohammad A. M. Eisa, Mohammed Alqurashi, Mohamed Sharaf, Nadi Awad Al-Harbi, Salem Mesfir Al-Qahtani, Mohamed F. M. Ibrahim

**Affiliations:** 1 Department of Agricultural Botany, Faculty of Agriculture, Ain Shams University, Cairo 11566, Egypt; asmaa.ahmed@agr.asu.edu.eg (A.A.S.-E.); mohamed_eisa@agr.asu.edu.eg (M.A.M.E.); 2 Department of Biology, College of Science, Princess Nourah bint Abdulrahman University, P.O. Box 84428, Riyadh 11671, Saudi Arabia; kmalwateed@pnu.edu.sa; 3 Horticulture Department, Faculty of Agriculture, Ain Shams University, Cairo 11566, Egypt; ahmed_abdelhafez2@agr.asu.edu.eg; 4 Agricultural Biotechnology Department, College of Agriculture and Food Sciences, King Faisal University, Al-Ahsa 31982, Saudi Arabia; 5 Biochemistry Department, Faculty of Agriculture, Cairo University, Giza 12613, Egypt; 6 Biology Department, Faculty of Science, University of Tabuk, Tabuk 71491, Saudi Arabia; balharbi@ut.edu.sa; 7 Department of Biotechnology, Faculty of Science, Taif University, Taif 21974, Saudi Arabia; m.khader@tu.edu.sa; 8 Department of Biochemistry, Faculty of Agriculture, AL-Azhar University, Cairo 11651, Egypt; mohamedkamel@azhar.edu.eg; 9 Department of Biochemistry and Molecular Biology, College of Marine Life Sciences, Ocean University of China, Qingdao 266003, China; 10 Biology Department, University College of Tayma, University of Tabuk, Tabuk 47512, Saudi Arabia; nalharbi@ut.edu.sa (N.A.A.-H.); salghtani@ut.edu.sa (S.M.A.-Q.)

**Keywords:** silicon, water stress, deficit irrigation, nanoparticles, gene expression, antioxidant enzymes

## Abstract

Recently, the use of nanofertilizers has received a great deal of attention in managing plants under biotic and abiotic stresses. However, studies that elucidate the role of silicon dioxide nanoparticles (SiO_2_NPs) in regulating maize tolerance to drought stress are still at early stages of development. In this study, plants that were treated with SiO_2_NPs (0.25 g/L as foliar spray) displayed considerable improvement in the growth indices, despite being subjected to drought stress. In addition, the action of SiO_2_NPs led to a considerable rise in the levels of chlorophylls, proline, cell membrane integrity, leaf water content, and antioxidant enzymes (superoxide dismutase (SOD), catalase (CAT), and guaiacol peroxidase (G-POX)). In contrast, an inverse trend was seen in the oxidative injury, the total amount of soluble sugars, and the activity of ascorbate peroxidase (APX). At the same time, carotenoids were unaffected in SiO_2_NPs-treated and non-treated plants under drought stress. The results of the molecular investigation that was conducted using qRT-PCR showed that the relative expression of the D2 protein of photosystem II (*PsbD*) was elevated in SiO_2_NPs-treated plants in response to drought stress, while the expression of the osmotic-like protein (*OSM-34*) and aquaporin (*AQPs*) was downregulated in SiO_2_NPs-treated plants in response to drought stress. This research could pave the way for further investigations into how SiO_2_NPs boost plant resistance to drought stress.

## 1. Introduction

Fighting climate change has become a global priority in recent years, due to the destructive consequences of climate change for the environment, the international economy, and food security [1,2]. In this context, the risk of drought stress has been observed in many regions worldwide [3,4]. Annually, drought stress can cause more severe losses in crop yields than all pathogens [5]. Physiologically, under drought stress, plants consume a great deal of adenosine triphosphate (ATP), which depletes their energy and results in irreparable harm or even plant death [6]. Moreover, drought stress causes severe decreases in cell division [7] and CO_2_ assimilation [8,9] and disturbs plant–water relations [10]. As the key organelles that carry out photosynthetic activities, the chloroplasts are expected to undergo alterations in their physiology and their protein pools as a result of drought-stress-induced fluctuations in leaf–gas exchanges and the buildup of reactive oxygen species (ROS) [11]. Changes to the expression levels of D2 protein (*psbD*), osmotin-like protein (*Osmotin-34*), and aquaporin (AQPs) seem to be essential regulating steps to induce tolerance mechanisms to drought stress [12,13,14].

*Zea mays* L., commonly known as maize, is the third most important cereal crop cultivated globally [15]. It is considered a rich energy source for humans and animals, due to its composition of carbohydrates, protein, fats, and vitamins [16]. Moreover, maize provides significant raw materials for multiple industries, including those that produce starch, feed, silage, and biofuels [17,18,19]. The production of maize in various regions of the world is significantly constrained by water stress, which is considered one of the most restrictive factors [8].

Recently, the use of nanoscale agrochemicals, including nano pesticides, nanoherbicides, and nanofertilizers, has received a great deal of attention [20]. It has been noticed that nanoparticles (NPs) are highly effective at enhancing the efficiency of water usage [21], controlling plant diseases [22], and combating various abiotic stresses [23]. In addition, the application of NPs is essential to ensure sustainable agriculture and reduce environmental pollution, including the treatment of wastewater and the removal of heavy metals [24,25].

The element Silicon (Si) has been found to have a positive impact on the growth and productivity of cereal crops, such as wheat [26], sugarcane [27], barley [28], and maize [29,30]. However, Si is an inert element with low solubility; therefore, the uptake of Si by plants is challenging [31]. The uptake of Si by plants has been enhanced using nanosilica, which is typically produced from bulk silica [32]. Nanosilica particles (Si-NPs) have a large surface-to-volume ratio, with a high uptake ability. These properties make them an optimal alternative to conventional chemical fertilizers [33]. It has been discovered that applying Si-NPs to plants may improve both their growth and their resistance to biotic and abiotic stresses [33,34]. Under drought stress, Si-NPs have been found to stimulate the synthesis of osmolytes and to induce antioxidative defense systems, leading to the mitigation of the effect of drought stress in barley [35]. In addition, treatment with Si-NPs can encourage maize plants to uptake more N, K, Cu, Mn, and Si under water deficiency [36]. The positive impacts of applied Si-NPs can also include the improvement of leaf pigments, photosynthesis, and stomatal conductance under drought stress [37,38].

This study was conducted to evaluate the role of SiO_2_NPs as a foliar spray in alleviating the deleterious effects of drought stress on maize seedlings, by observing a wide spectrum of aspects which SiO_2_NPs can induce tolerance to drought stress at morphological, physiological, biochemical, and molecular levels.

## 2. Results and Discussion

### 2.1. Characterization of SiO_2_

#### 2.1.1. XRD Patterns

**Figure 1:**
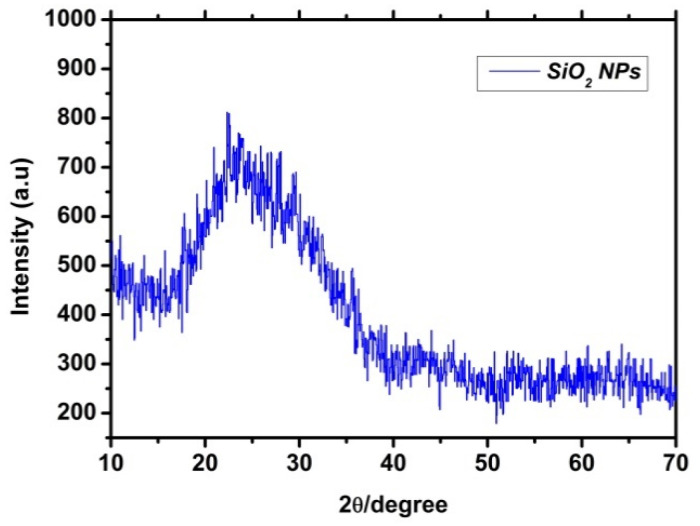
X-ray diffraction patterns of SiO_2_ nanoparticles.

The bare SiO_2_ nanoparticle X-ray diffraction patterns are seen in (Figure 1). The characteristic peak at 23° (2θ) for the NPs sample may be used to prove the amorphous nature of the substance [39].

#### 2.1.2. Surface Morphology by TEM

**Figure 2:**
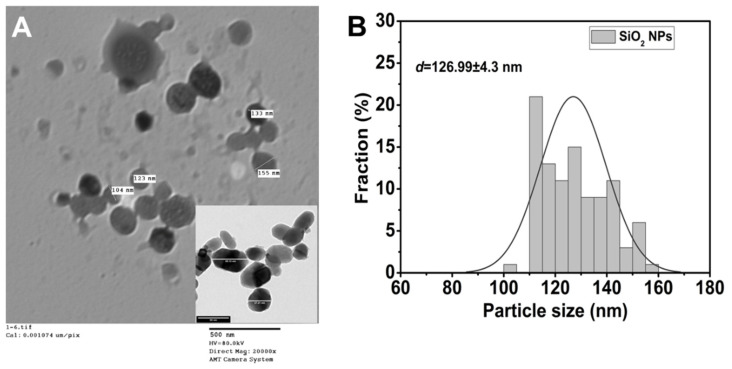
Surface morphology of SiO_2_ nanoparticles (**A**) TEM image, (**B**) particle size distribution.

In this study, we described the creation of SiO_2_ nanoparticles using the sol–gel method and investigated their morphological structure using TEM. The results are displayed in Figure 2A,B. The TEM image and particle size distribution of the SiO_2_ nanoparticles produced under various sol–gel conditions are shown in Figure 2A. The homogeneous size distribution and spherical shape of the SiO_2_ nanoparticles can be seen in the image. Additionally, TEM analysis showed that the nanoparticles had negligible agglomeration. The particle size range was determined to be approximately ~126 nm in Figure 2B, which virtually indicated a tight size distribution [40,41]. Therefore, the optical and structural properties of SiO_2_ nanoparticles can be tailored by using synthesis process parameters for the plant stress application.

### 2.2. Effect of SiO_2_NPs Application on Growth

**Figure 3:**
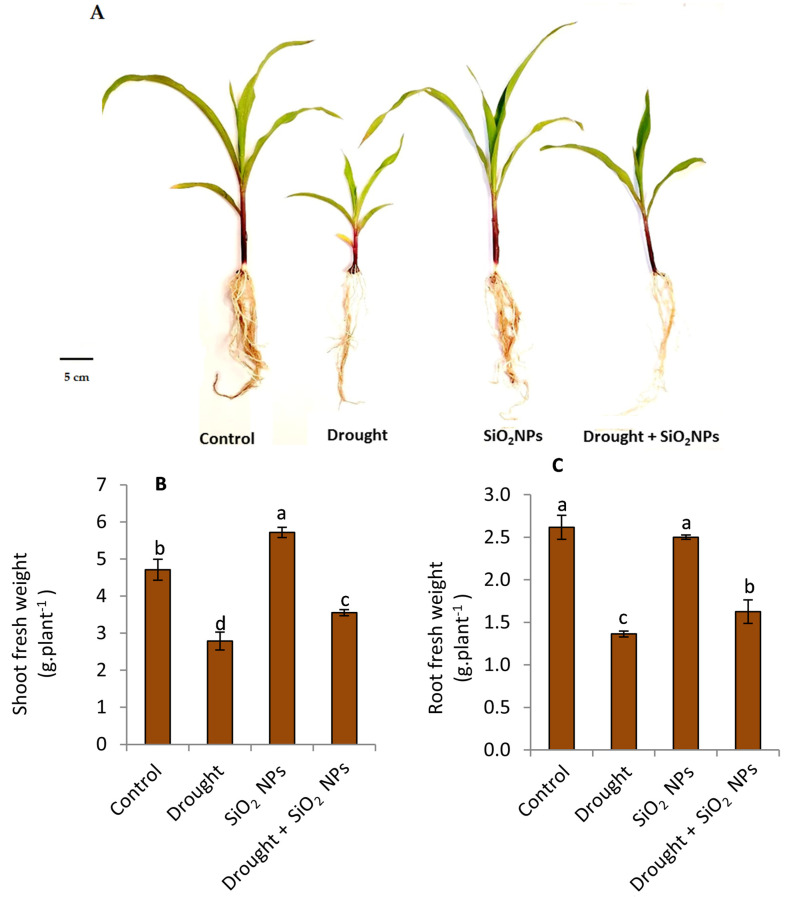
Effect of applied SiO_2_-NPs on the vegetative growth (**A**), shoot fresh weight (**B**) and root fresh weight (**C**) of maize seedlings under normal irrigation and drought stress. The data that have been presented with a ±SD notation represent the mean values of three separate replicates. Significant variations were observed based on Duncan’s multiple range test at a significance level of *p* ≤ 0.05, as indicated by the distinct lowercase letters.

The results in Figure 3 indicate that the shoot and root fresh weights have decreased significantly due to drought stress compared to normal irrigation conditions. Furthermore, the application of SiO_2_NPs has resulted in a noteworthy augmentation in the shoot fresh weight of maize seedlings in comparison to the seedlings that were not subjected to treatment under standard or drought conditions (Figure 3B). Under normal conditions, the application of SiO_2_NPs has no effect on root fresh weight; under drought conditions, SiO_2_NPs application improved root fresh weight (Figure 3C).

The most serious abiotic stress on crops is drought, which is brought on by insufficient rainfall and/or irregular precipitation patterns. The role of Si in improving vegetative growth has been reported in previous works such as that of Gong et al. [42] on wheat and that of Shen et al. [43] on soybean. Also, our results indicate that SiO_2_NPs improved shoot fresh weight under stress conditions or normal conditions (Figure 3B). In accordance with our results in Figure 3C, previous works reported the enhancement of root growth and weight by SiO_2_ application in Sorghum under drought stress [44,45]. This result could be due to the role of SiO_2_ in promoting water uptake under drought stress by controlling osmotic adjustment and modifying the expression proteins controlling the water channel [46]. In addition, the role of Si in increasing root length to recover from water stress was reported [47]. The role of SiO_2_NPs in improving the shoot and root growth of maize seedlings may be due to the role of Si in stimulating the process of photosynthesis, which leads to an increase in growth [48] or decreasing stomatal transpiration [49]. Also, Si treatment under drought stress could increase the fresh weight of maize seedlings by increasing water and osmotic potential and maintaining higher turgor pressure in plants [50].

### 2.3. Effect of SiO_2_NPs Application on Photosynthesis Pigments

**Figure 4:**
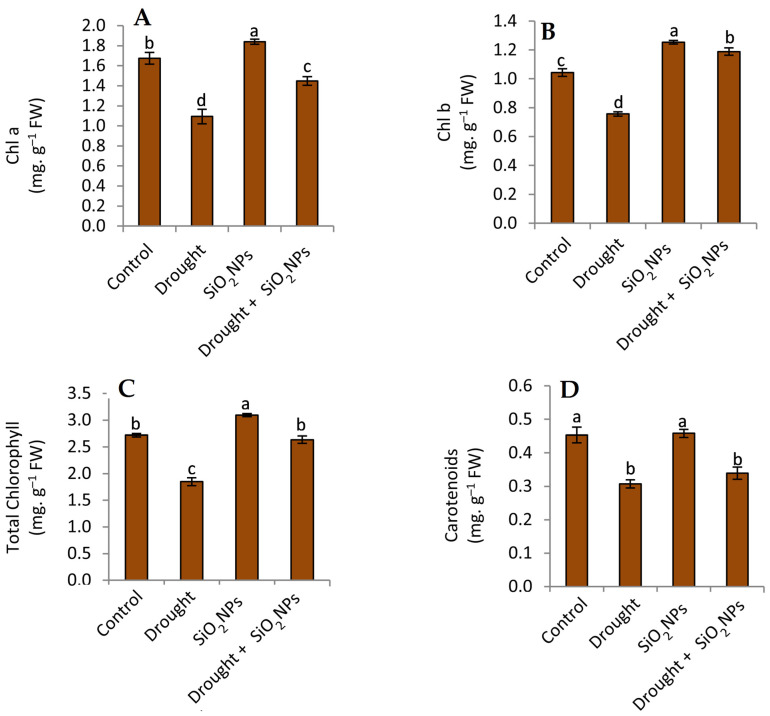
Effect of applied SiO_2_NPs on photosynthetic pigments. (**A**) Chlorophyl a, (**B**) chlorophyll, (**C**) total chlorophyll and (**D**) carotenoids of maize seedlings under normal irrigation and drought stress. The data that have been presented with a ±SD notation represent the mean values of three separate replicates. Significant variations were observed based on Duncan’s multiple range test at a significance level of *p* ≤ 0.05, as indicated by the distinct lowercase letters.

The results in Figure 4 indicate that the content of photosynthesis pigments in the maize seedlings was higher in the control treatment (non-water stress) compared with the drought stress condition. In addition, the treatment of maize seedlings with SiO_2_NPs has led to a significant increase in all previous compounds (except carotenoids) compared to seedlings that have not been treated. Under conditions of water stress, the application of SiO_2_NPs resulted in a noteworthy augmentation of chlorophyll a, chlorophyll b, and total chlorophyll in maize seedlings (Figure 4A–C). The study found that there was no statistically significant disparity in the carotenoid composition of seedlings subjected to SiO_2_NPs treatment and those that were not treated (as illustrated in Figure 4D), in the presence of water stress conditions. Reduced photosynthesis is one of the main effects of drought, which results from decreased leaf growth, damaged photosynthetic machinery, early leaf senescence, and a corresponding decline in food production [51]. This study confirms that drought stress reduces photosynthetic pigments (Figure 4A–D). This result could be due to stomatal and non-stomatal photosynthetic limitations under drought stress conditions [52]. Epstein [31] has reported an elevation in the enzymatic activity responsible for chlorophyll degradation, while Mafakheri et al. [53] have highlighted the adverse impact of ROS on chloroplast. Since chlorophyll content is positively correlated with the rate at which plants produce biomass through photosynthesis, it significantly determines plant productivity [54]. In harmony with our findings, Si treatment has been found to improve the photosynthetic rate under abiotic stress and increase chlorophyll pigments in wheat [44] and soybean [43]. It is well known that Si regulates the stomatal function and might affect photosynthetic efficacy by affecting the relations of gas exchange [55]. Also, Si application often enhances stomatal conductance (Kang et al., 2016) and leads to an increase in photosynthetic rates [56].

### 2.4. Effect of SiO_2_NPs Application on RWC, Proline and Total Soluble Sugars

**Figure 5:**
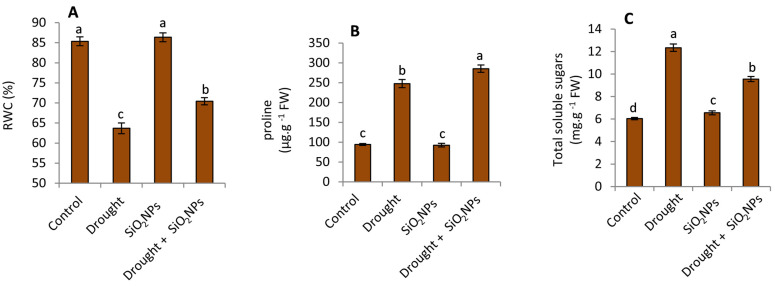
Effect of applied SiO_2_-NPs on (**A**) leaf relative water content, (**B**) proline, and (**C**) total soluble sugars of maize seedlings under normal irrigation and drought stress. The data that have been presented with a ±SD notation represent the mean values of three separate replicates. Significant variations were observed based on Duncan’s multiple range test at a significance level of *p* ≤ 0.05, as indicated by the distinct lowercase letters.

The data depicted in Figure 5 indicate that when subjected to drought stress, there was a notable reduction in relative water content (RWC). Conversely, there was a marked and statistically significant increase in proline and total soluble sugars levels in comparison to plants that were adequately watered. In contrast, the RWC and proline levels of the treated plants were much higher than those of the control plants. However, total soluble sugars displayed an opposite trend compared to the untreated plants under drought stress. Nanosilica was able to stimulate the antioxidative defense system and osmolyte accumulation, which reduced the severity of drought stress in barley [35], wheat [57], and [36] maize. It is well known that Si plays an important role in decreasing the transpiration rate under drought stress [58]. Moreover, Si deposits in the cell walls, particularly the xylem tissue, may be able to stop any compression that occurs in these vessels under drought stress [59]. The findings of this investigation suggest that the application of SiO_2_ nanoparticles may have a significant impact on the tolerance of maize seedlings to drought stress. Specifically, the observed increase in proline levels and decrease in total soluble sugars following SiO_2_NPs treatment can play a crucial role in this regard. These results highlight the potential of SiO_2_NPs to facilitate osmotic adjustment, with particular emphasis on the modulation of proline accumulation over the accumulation of soluble sugars.

### 2.5. Effect of SiO_2_NPs Application on H_2_O_2_, MDA, and CMSI

**Figure 6:**
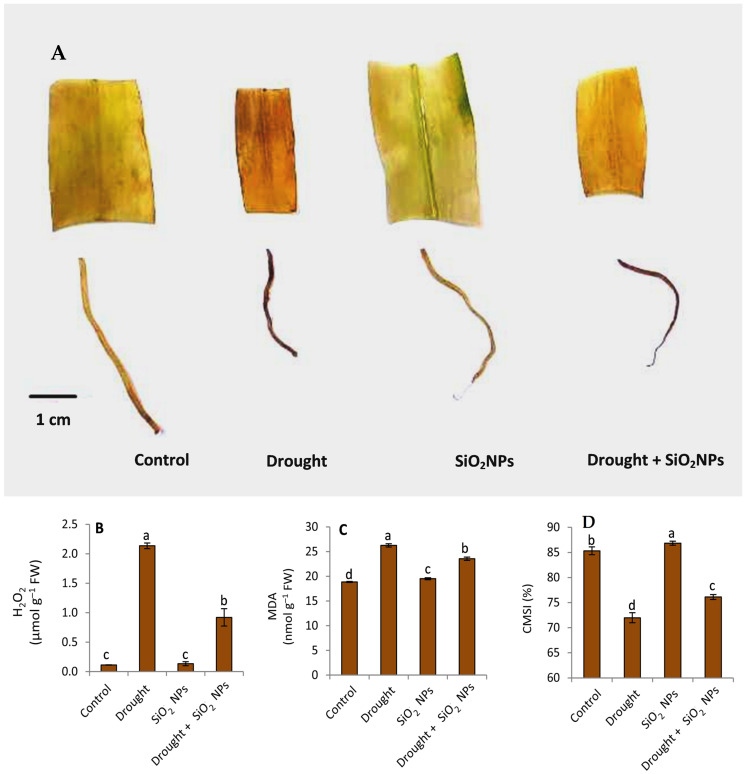
Effect of applied SiO_2_NPs on (**A**) the histochemical detection of H_2_O, (**B**) H_2_O_2_ concentration, (**C**) malondialdehyde MDA and (**D**) cell membrane stability index CMSI of maize seedlings under normal irrigation and drought stress. The data that have been presented with a ±SD notation represent the mean values of three separate replicates. Significant variations were observed based on Duncan’s multiple range test at a significance level of *p* ≤ 0.05, as indicated by the distinct lowercase letters.

The results in Figure 6A,B show that the content of H_2_O_2_ in maize seedlings has significantly increased under drought conditions compared with seedlings grown under normal irrigation conditions. In addition, the treatment with SiO_2_NPs did not affect the H_2_O_2_ content of the seedlings under normal irrigation conditions. On the other hand, a discernible beneficial impact was observed in mitigating the concentration of hydrogen peroxide (H_2_O_2_) in plants treated with silicon dioxide nanoparticles (SiO_2_NPs) during instances of water stress. The results in Figure 6C indicate that the malondialdehyde (MDA) content in maize seedlings increased when seedlings were subjected to drought conditions compared to normal irrigation conditions. Furthermore, the application of SiO_2_ nanoparticles resulted in a noteworthy reduction in malondialdehyde levels in comparison to non-treated vegetation exposed to drought conditions. As expected, the percentage of CMSI decreased in maize seedlings grown under normal irrigation conditions compared to those under drought stress Figure 6D. The SiO_2_NPs application increased the percentage of CMSI under both water conditions.

Hydrogen peroxide is a signaling agent that crosses cell membranes, especially for stress adaptation and antioxidant defense [60]. According to our findings, other studies have also indicated that Si can lower MDA and H_2_O_2_ levels [42,61,62]. Si’s role in mitigating stress conditions such as drought could be due to its role in controlling the expression of genes such as TaSOD, TaCAT, and TaAPX137 that are involved in synthesizing and activating antioxidant enzymes [63]. Several factors, such as time of occurrence, duration, and severity, influence the harmful effect of drought on crops. An important physiological guide for assessing drought tolerance is CMSI, which is inversely correlated with cell membrane damage [64]. Furthermore, the findings depicted in Figure 3C indicate a reduction in CMSI in response to water stress. Similarly, Maghsoudi et al. [65] reported decreased CMSI in wheat plants under drought stress. Additionally, they found that Si application enhanced the CMSI under both irrigation conditions.

### 2.6. Impact of SiO_2_NPs Administration on Antioxidant Enzymes

**Figure 7:**
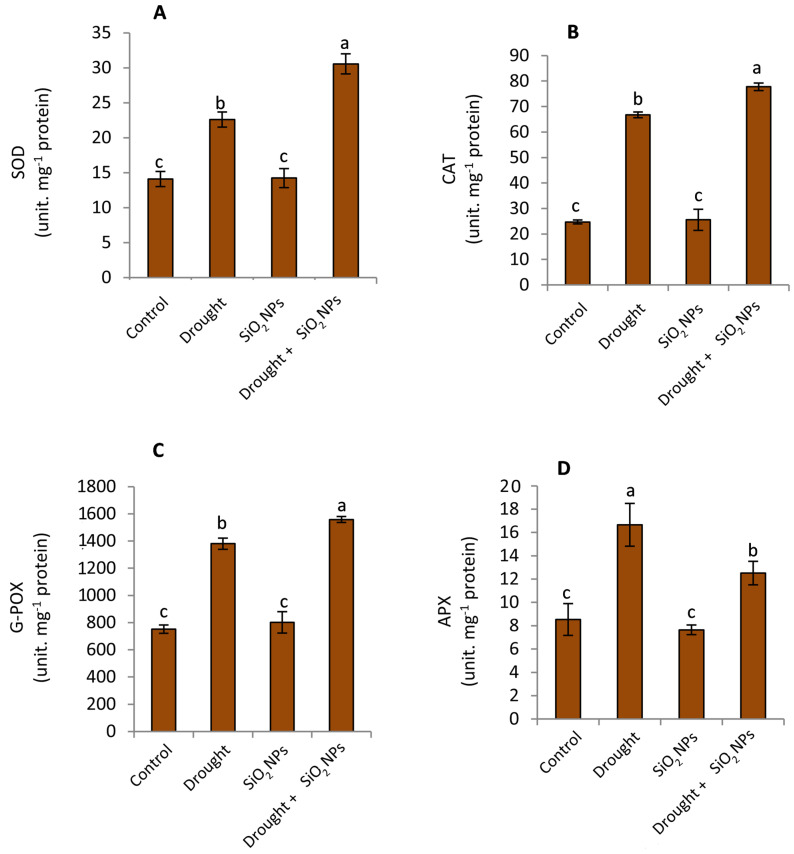
Effect of applied SiO_2_NPs on activities of (**A**) superoxide dismutase SOD, (**B**) catalase CAT, (**C**) peroxidase POX, and (**D**) ascorbate peroxidase APX of maize seedlings under normal irrigation and drought stress. The data that have been presented with a ±SD notation represent the mean values of three separate replicates. Significant variations were observed based on Duncan’s multiple range test at a significance level of *p* ≤ 0.05, as indicated by the distinct lowercase letters.

As anticipated, the enzymatic activities of SOD, CAT, G-POX, and APX have exhibited a rise in response to water stress conditions in contrast with the standard irrigation conditions, as depicted in Figure 7A–D. The application of SiO_2_NPs resulted in an increase in the activity of all enzymes studied, with the exception of APX, when subjected to water stress conditions. Conversely, there was no notable disparity in enzyme activity under normal irrigation conditions.

The impact of abiotic stresses on equilibrium between reactive oxygen species (ROS) and antioxidants has been well documented, resulting in oxidative impairment of cellular membrane architecture [66,67,68,69]. By boosting plant defensive responses, such as those of the antioxidant system, Si treatment improves the resilience and tolerance of plants to drought stress, hence reducing oxidative stress brought on by drought [70]. Prior studies have indicated that the utilization of silicon augmented the efficacy of antioxidant enzymes in particular crops, such as wheat and tomatoes, under conditions of abiotic stress [44,71]. Superoxide dismutase (SOD) serves as the primary defense mechanism against reactive oxygen species (ROS) by catalyzing the conversion of superoxide or singlet oxygen radicals into molecular oxygen and hydrogen peroxide [72]. SOD’s role in the defense mechanism responsible for neutralizing oxidative stress is clearly indicated by the rise in cellular SOD activity when there is environmental stress, such as drought [73]. By collaborating with SOD to remove ROS and break down H_2_O_2_ into water and oxygen, CAT serves a beneficial role [74]. In addition, MDHAR is synthesized in multiple cellular compartments through the enzymatic conversion of H_2_O_2_ to H_2_O by APX, which employs ascorbate as a hydrogen donor. The primary enzyme removes H_2_O_2_ from plant cells’ chloroplasts [75]. Previous studies demonstrated, in a manner that is in agreement with our findings, the involvement of Si application in enhancing the activity of antioxidant enzymes in the presence of abiotic stressors [71,76,77,78]. SOD, CAT, and POX activity also rose in wheat under drought stress, but more so in the presence of Si, according to Sattar et al. [55]. The observed outcomes may be attributed to the significant augmentation of antioxidant enzymes APX and G-POX due to the amplification of the ascorbic acid–glutathione cycle and related enzymes by Si [79].

### 2.7. Effect of SiO_2_NPs Application on Gene Expression

**Figure 8:**
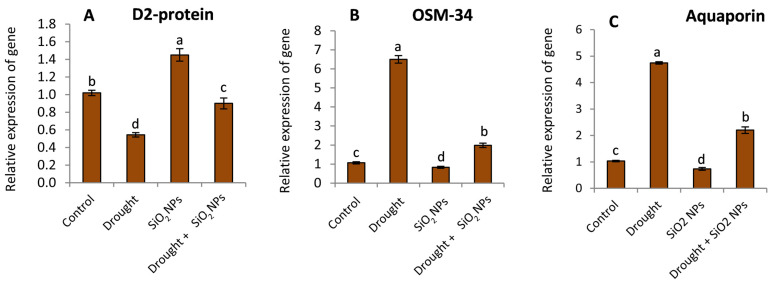
Effect of applied SiO_2_NPs on relative expression of (**A**) D2 protein, (**B**) OSM-34, and (**C**) Aquaporin of maize seedlings under normal irrigation and drought stress. The data that have been presented with a ±SD notation represent the mean values of three separate replicates. Significant variations were observed based on Duncan’s multiple range test at a significance level of *p* ≤ 0.05, as indicated by the distinct lowercase letters.

The results in Figure 8A indicate that expression of the D2 protein gene decreased under drought stress compared with regular irrigation. Moreover, the D2 protein expression was increased by SiO_2_ NPs application under drought conditions, while the D2 protein gene was significantly increased under normal water conditions. The expressions of both OSM-34 and aquaporin genes were found to be upregulated in response to drought stress compared to well-watered conditions (Figure 8B,C). Moreover, the treatment with NPsSiO_2_ led to a decrease in the expression of OSM-34 and Aquaporin genes under drought and normal conditions compared to the untreated plants.

In agreement with our results, Si treatment elevated the expression of genes related to oxidative stress [63]. Some previous works suggested that gene expression may be impacted by Si fertilization [80,81]. In this study, plants treated with SiO_2_NPs seem to impact gene expression. D2 protein is placed as a core for photosystem II inside the plastid; thus, it is important to the integrity of thylakoid membranes and the efficiency of photosynthesis [82]. Under stressful circumstances, D2 protein is susceptible to oxidative damage and photoinhibition [83]. These effects can explain the downregulation of the expression of the D2 protein under water stress. Meanwhile, the role of SiO_2_NPs in safeguarding the machinery of photosynthesis under drought stress may be reflected in their favorable effect on the expression of D2 protein.

Aquaporins (AQPs), which are widely recognized membrane channel proteins, have the ability to transport water, metal ions, gases, and small neutral solutes in reaction to both biotic and abiotic stressors [84]. Numerous factors, like phosphorylation, cytosolic pH, divalent cations, reactive oxygen species, and stoichiometry, are frequently employed to regulate aquaporin gating. Hydraulic conductance, root system architecture, abiotic stress-related gene modulation, seed viability and germination, phloem loading, xylem water exit, photosynthetic parameters, and post-drought recovery have all been connected to them [14].

Additionally, a cysteine-rich protein called osmotin (OSM-34) is created in vacuoles and acts as an osmoregulator when the water potential is low [85]. The overexpression of osmotin protein in plants can protect them from different stresses by reducing reactive oxygen species (ROS) production, limiting lipid peroxidation, initiating programmed cell death (PCD), increasing proline content, and scavenging enzyme activity [12].

In this work, the reduction in AQPs and OSM-34 expression in plants treated with SiO_2_NPs suggests enhanced membrane and RWC integrity (Figure 5 and Figure 6) compared to SiO_2_NPs untreated plants under drought stress.

## 3. Materials and Methods

### 3.1. Plant and Experimental Details

This study used maize seeds of a single white hybrid (Egaseed 81) as plant material. Seeds were soaked in a solution of 0.1% sodium hypochlorite for 5 min, followed by four rounds of washing. Subsequently, the seeds were subjected to incubation at 25 °C for 48 h, in the absence of light. The seeds were placed on moist filter paper and provided with distilled water. Following the emergence of radicals, four seedlings that exhibited similar growth patterns were chosen and subsequently transplanted into black plastic pots measuring 20 cm in diameter and 25 cm in height. A total of 48 pots were maintained in a greenhouse environment, with an average diurnal temperature of 28.2 ± 3.6 °C and an average nocturnal temperature of 19.6 ± 2.8 °C. The relative humidity was 64.5 ± 4.9%, and the duration of natural daylight ranged from 11 to 12 h. Every two days, tap water and a half-strength Hoagland’s solution were used to water all pots [86]. The volume of water, or Hoagland’s solution, was calculated directly using the weight method to maintain soil moisture at 65–75% of the field capacity.

### 3.2. Experimental Design and Treatments Organization

**Figure 9:**
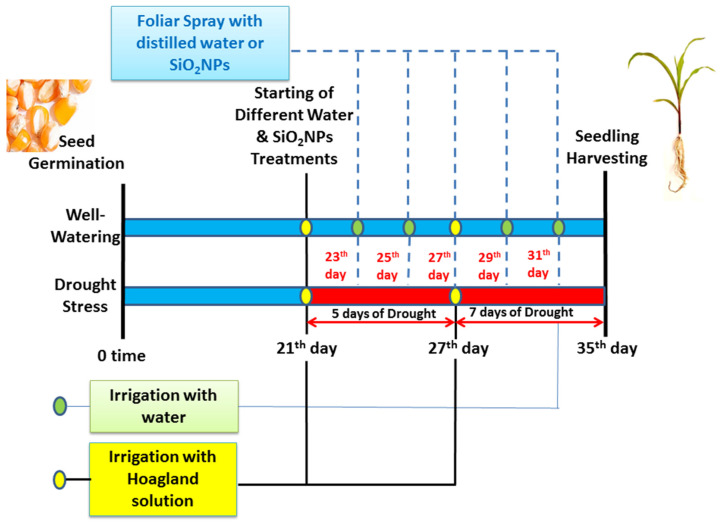
A simplified model shows the different treatments (irrigation and foliar applications with distilled water or SiO_2_NPs) and the sampling date of maize seedlings.

Subsequently, after three weeks (three-true leaf stage), four treatments were applied as follows: (i) control; pots were well irrigated day by day (field capacity: 65–75% using the direct weight method) + foliar applications with distilled water, (ii) drought stress; irrigation was stopped in two successive periods (5 and 7 days) + foliar applications with distilled water; (iii) SiO_2_NPs; pots were well irrigated day by day + foliar applications with 0.25 g/L SiO_2_NPs and (iv) drought stress + SiO_2_NPs; irrigation was stopped in two successive periods (5 and 7 days) + foliar applications with 0.25 g/L SiO_2_NPs. The timeline infographic of various treatments (irrigation and foliar applications) and sampling is shown in Figure 9. All treatments were irrigated twice (at 21 and 27 days after sowing) with 1.5 L of half-strength Hoagland’s solution to give all plants in drought and well-watered treatments the same amounts of nutrients. A preliminary study was conducted to determine the optimum concentration of SiO_2_NPs by observing the decrease in the rate of lipid peroxidation under drought stress (Figure S1). All foliar treatments (distilled water or SiO_2_NPs) were sprayed five times with 20 mL of a fresh solution with 0.05% tween-20 at 23, 25, 27, 29, and 31 days after sowing. The total number of pots was 48 and distributed in a complete randomized design (CRD), including two foliar treatments X 2 watering levels X 4 pots X 3 replicates. The maize seedlings were permitted to continue growing for an additional three days following the final foliar application. Subsequently, samples were gathered to evaluate growth and various biochemical constituents.

### 3.3. Experimental Approach and SiO_2_ Preparation

Tetraethyl orthosilicate (TEOS) (Si(OC_2_H_5_)), acetic acid (CH_3_COOH), methyl acetate (C_3_H_6_O_2_), and methanol (CH_3_OH) were among the chemicals acquired from Sigma-Aldrich as beginning ingredients. Silicon dioxide (SiO_2_) nanoparticles were synthesized via the sol–gel method, according to Saravanan and Dubey [87]. At the outset, a mixture of 2.3 mL of acetic acid and 20 mL of methanol was blended and agitated for 5 min at ambient temperature. The water molecules partially evaporated, resulting in the production of methyl acetate. During the same period, 1.5 mL more of TEOS was added drop by drop. Using vigorous stirring for 90 min, a homogenous translucent solution was produced.

*CH_3_COOH + CH_3_OH → C_3_H_6_O_2_ + H_2_O*↑*C_3_H_6_O_2_ + Si (OC2H5)4 → SiO_2_ + C_11_H2_6_ O_4_*↑

At room temperature, the produced SiO_2_ NPs solution was then dried. To create fine nanoparticles, the dried SiO_2_ product was ground, calcined at 500 °C, and then grained. The produced samples were analyzed using TEM and XRD after calcination.

### 3.4. Characterization of SiO_2_

#### 3.4.1. Phase Development Determination

The development of the hydrated phase at various curing ages and the mineralogical makeup of the basic ingredients were determined using XRD. The pastes’ XRD patterns were examined using Cu Kα radiation at 0.154 nm on a Rigaku SmartLab 3000Å diffractometer (Tokyo, Japan).

#### 3.4.2. Transmission Electron Microscopy (TEM)

TEM was employed in order to investigate the shape of the SiO_2_NPs as well as their dispersion by placing 10 μL of diluted sample onto holey carbon films on copper grids. Samples were observed operating at an accelerating voltage of 200 kV. Nanoparticle size was measured using ImageJ software (version 1.52a).

### 3.5. Determination of Growth Parameters

The shoot and root fresh weight at the 35-day-old stage was recorded immediately using a digital balance.

### 3.6. Determination of Photosynthetic Pigments

The photosynthetic pigments of the maize leaves were determined according to Yang et al. [88]. Using a mortar and pestle and 80% acetone, the fresh leaves (0.1 g) were extracted. Spectrophotometers measured the solution’s absorbance at different wavelengths. The expression for photosynthetic pigments was expressed as mg g ^−1^ FW.

Chl a (μg/mL)= 12.25 A_663.6_ − 2.55 A_646.6_

Chl b (μg/mL)= 20.31 A_646.6_ − 4.91 A_663.6_

Chl Total (μg/mL)= 17.76 A_646.6_ + 7.34 A_663.6_

Car (μg/mL)= 4.69 A_440.5_ − 0.267 Chl Total

### 3.7. Histochemical Detection of H_2_O_2_

To investigate the existence of H_2_O_2_ histochemically using the diaminobenzidine (DAB) method [89], a part of the ditched leaf from each treatment was completely soaked in a Petri dish containing a solution of 100 ppm DAB and 50 mM Tris-HCl buffer, pH 4.0, for 24 h. After that, the leaf pieces were transferred to absolute alcohol several times to remove the leaf pigment. The buildup of H_2_O_2_ in leaf tissues grew along with the appearance of a dark brown color.

### 3.8. Quantification of H_2_O_2_ and Lipid Peroxidation

According to Velikova et al. [90], with a few adjustments, the hydrogen peroxide (H_2_O_2_) content was determined. In tri-chloroacetic acid (TCA), leaf samples (0.2 g) were homogenized. At 10,000 rpm and 4 °C for 10 min, the homogenate was centrifuged. Then, 0.75 mL of the supernatant was added to 1.5 mL of 1 M KI and 0.75 mL of 10 mM K-phosphate buffer (pH 7.0). By comparing H_2_O_2_’s absorbance at 390 nm, the concentration of H_2_O_2_ was determined.

Malondialdehyde (MDA) measurement was used to measure lipid peroxidation, as explained by Heath and Packer [91]. The fourth leaf from the top was used to homogenize leaf tissues with 0.1% (*w*/*v*) trichloroacetic acid (TCA). For 15 min, the homogenate was centrifuged at 4500 rpm. Meanwhile, 1 mL of the supernatant and 4 mL of 0.5% (*w*/*v*) thiobarbituric acid (TBA) mixed in 20% (*w*/*v*) TCA made up the reaction mixture. The mixture was heated for 30 min in boiling water, cooled to room temperature, and then centrifuged for 15 min at 4500 rpm. The absorbance (A) of the supernatant was measured at 535 nm and corrected for non-specific turbidity at 600 nm using a spectrophotometer. The MDA concentration (nmol g^−1^ FW) was calculated using Δ OD (A532-A600) and the extinction coefficient (ε = 155 mM^−1^ cm^−1^)

### 3.9. Determination of Cell Membranes Stability Index (CMSI)

The method employed to ascertain the stability of the cell membrane was based on the protocol outlined by González and González-Vilar [92], albeit with slight modifications. Specifically, ten leaf discs with a diameter of 1 cm were immersed in 10 mL of deionized water and subjected to agitation for 24 h. The EC1 readings were recorded using an EC meter. Subsequently, all specimens underwent a thermal treatment in boiling water for 20 min, following which the measurements were once again documented (EC2).

(1) $$MSI=\left[1-\left(\frac{EC1}{EC2}\right)\right]\times 100$$

### 3.10. Determination the Activities of Antioxidant Enzymes

Fresh leaf samples were combined with a phosphate buffer (5 mL, 50 mM, 7.8 pH) and centrifuged at 6000 rpm for 20 min. Inhibiting NBT (nitroblue tetrazolium) reduction is the fundamental method for calculating superoxide dismutase (SOD) activity at 560 nm [93]. The primary reactants in this reaction were 1 mL of NBT (50 µM), 1 mL of riboflavin (0.5 mM), 50 µL of enzyme extract, 900 µL of phosphate buffer (50 mM), and 50 µL of methionine (13 mM). The mixture was first exposed to 30 W of fluorescent lamp light to begin the reaction. The reaction ceased when the lamp was switched off after five minutes. The blue formazan produced via NBT reduction was visible at 560 nm. The identical reactants were used to take a blank reading, but no enzyme extract was present. The Aebi [94] methodology was employed to quantify the catalase (CAT) activity. The decline in absorbance at 290 nm of ascorbate oxidation was used to determine the activity of ascorbate peroxidase (APX) [95]. Guaiacol peroxidase (G-POX) activity was assayed by measuring guaiacol’s oxidation by observing the absorbance increase at 470 nm for 3 min [96].

### 3.11. Relative Water Content (RWC), Total Soluble Sugars, and Proline

Leaf discs (1 cm in diameter) were used to measure RWC in leaves [8]. The leaf discs were floated on distilled water in the dark for five hours after each disc’s fresh weight (FW) measurement to obtain turgid weights (TW). The RWC in leaves was determined using leaf discs (1 cm diameter) [97]. Total soluble sugar was measured utilizing the method described by Chow and Landhäusser [98], with some modifications. Proline content was evaluated as per Bates et al.’s [99] methodology.

### 3.12. Gene Expression

The mRNA from each treatment (0.5 g of leaves) was isolated utilizing an RNA extraction kit (Sigma-Aldrich, St. Louis, MO, USA). Following reverse transcription and cDNA formation, the primer sequences (Table 1) used in real-time PCR with SYBR^®^ Green and GAPDH were used as housekeeping genes. Relative gene expression was determined using 2^−ΔΔCt^ [100].

### 3.13. Statistics

The SAS [101] software was utilized to conduct the one-way ANOVA procedure. The results of three replicates were presented as varying values ± standard deviation. The differences between means were determined according to Duncan’s multiple range test.

## 4. Conclusions

Silica nanoparticles have been identified as a potentially effective means of improving plant growth and yield and mitigating other stresses. However, these techniques are still in an early stage of development. Therefore, this study has tried to focus on how the nanosilica mediates the tolerance mechanisms to drought stress in maize seedlings. The results confirmed that SiO_2_NPs could improve growth and trigger several strategies to combat the detrimental effects of drought stress. The responses observed encompassed enhancements in photosynthetic pigments, plant hydration levels, osmolyte accumulation, heightened activity of antioxidant enzymes, mitigation of oxidative injury, and mediation of gene expression regulation. SiO_2_NPs can be recommended as an excellent alternative to conventional chemical fertilizers. However, future studies should focus on the concentrations of SiO_2_NPs that can lead to phytotoxicity and their long-term effects on the environment. Further molecular studies should evaluate the underlying mechanisms behind various biochemical pathways of secondary metabolites in plants.

## Figures and Tables

**Table 1:** Sequences of primers utilized for quantitative RT-PCR analysis.

Gene Name	Sequence		Accession Number
*Osmotin-like protein* (*Osmotin-34*)	F	5′-GAACGGAGGGTGTCACAAAATC-3′	NM_001153626.2
	R	5′-CGTAGTGGGTCCACAAGTTCCT-3′	
*D2 protein (psbD)*	F	5′- GGAAGATCAATCGACCGAAA-3′	S46931.1
	R	5′- CCTTATGCACCCATTTCACA-3′	
*Aquaporin* (*AQPs*)	F	5′- GTTCCTATCCTTGCCCCACT-3′	AY243801.1
	R	5′- AGGCGTGATCCCTGTTGTAG-3′	
*GAPDH* (The housekeeping gene)	F	5′- TTGGTTTCCACTGACTTCGTT-3′	X15596.1
	R	5′-CTGTAGCCCCACTCGTTGT-3′	

## Data Availability

Not applicable.

## Supplementary Materials

The following supporting information can be downloaded at: https://www.mdpi.com/article/10.3390/plants12142592/s1, Figure S1: Effect of different concentrations of water and SiO2NPs on the membrane lipid oxidation of maize seedlings grown under control and drought stress.
