# Supplementary material for: Response of Maize Seedlings to Silicon Dioxide Nanoparticles (SiO2NPs) under Drought Stress
Source: Plants (Basel). 2023 Jul 8;12(14):2592. doi: 10.3390/plants12142592 (PMC10386567; doi:10.3390/plants12142592)
Supplement: Supplementary file 1 [file plants-12-02592-s001.zip › plants-2472348-supplementary.pdf]

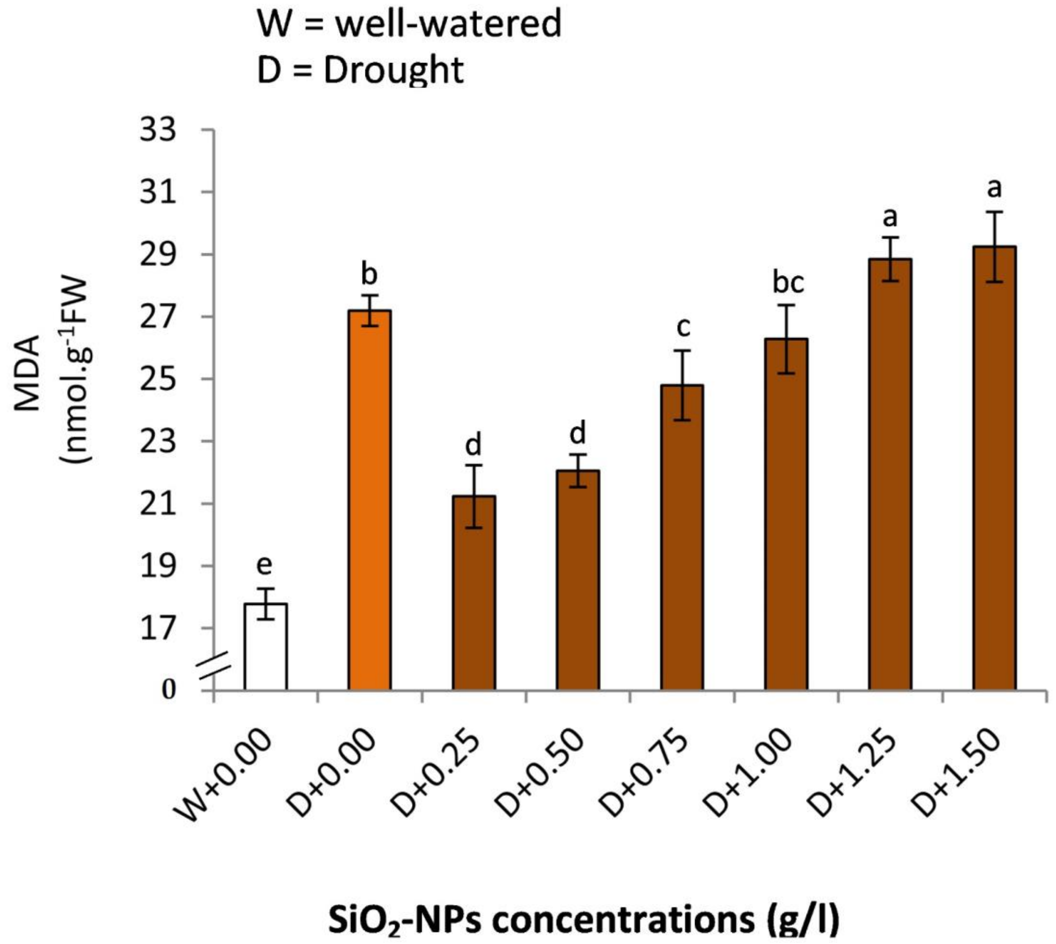

Figure S1. Effect of different concentrations of water and SiO_2NPs on the membrane lipid oxidation of maize seedlings grown under control and drought stress
